# Empirical Evidence on Enhanced Mutation Rates of 19 RM-YSTRs for Differentiating Paternal Lineages

**DOI:** 10.3390/genes13060946

**Published:** 2022-05-26

**Authors:** Faqeeha Javed, Muhammad Shafique, Dennis McNevin, Muhammad Usama Javed, Abida Shehzadi, Ahmad Ali Shahid

**Affiliations:** 1 Forensic Research Laboratory, Centre of Excellence in Molecular Biology, University of the Punjab, Lahore 53700, Pakistan; faqeeha.javed@cemb.edu.pk (F.J.); abida@cemb.edu.pk (A.S.); ahmadali.shahid@gmail.com (A.A.S.); 2 Centre for Forensic Science, University of Technology Sydney, Sydney 2007, Australia; dennis.mcnevin@uts.edu.au; 3 Faculty of Medicine, Allama Iqbal Medical College, University of Health Sciences, Lahore 54700, Pakistan; usamajaved0343@gmail.com

**Keywords:** RM-YSTRs, mutation rate, meiosis, paternal lineage, multiplex

## Abstract

Rapidly mutating Y-chromosomal short tandem repeats (RM Y STRs) with mutation rates ≥ 10^−2^ per locus per generation are valuable for differentiating amongst male paternal relatives where standard Y STRs with mutation rates of ≤10^−3^ per locus per generation may not. Although the 13 RM Y STRs commonly found in commercial assays provide higher levels of paternal lineage differentiation than conventional Y STRs, there are many male paternal relatives that still cannot be differentiated. This can be improved by increasing the number of Y STRs or choosing those with high mutation rates. We present a RM Y STR multiplex comprising 19 loci with high mutation rates and its developmental validation (repeatability, sensitivity and male specificity). The multiplex was found to be robust, reproducible, specific and sensitive enough to generate DNA profiles from samples with inhibitors. It was also able to detect all contributor alleles of mixtures in ratios up to 9:1. We provide preliminary evidence for the ability of the multiplex to discriminate between male paternal relatives by analyzing large numbers of male relative pairs (536) separated by one to seven meioses. A total of 96 mutations were observed in 162 meioses of father–son pairs, and other closely related male pairs were able to be differentiated after 1, 2, 3, 4, 5, 6 and 7 meiosis in 44%, 69%, 68%, 85%, 0%, 100% and 100% of cases, respectively. The multiplex offers a noticeable enhancement in the ability to differentiate paternally related males compared with the 13 RM Y STR set. We envision the future application of our 19 RM Yplex in criminal cases for the exclusion of male relatives possessing matching standard Y STR profiles and in familial searching with unknown suspects. It represents a step towards the complete individualization of closely related males.

## 1. Introduction

Y STRs are important genetic markers commonly employed for the detection of male DNA in a female DNA background, paternity testing and lineage searching [1]. Paternally related males typically share similar (if not the same) Y-STR haplotypes [2]. The differentiation of male relatives in forensic investigations is important, particularly among closely related males such as father/son pairs, brothers and cousins [1]. It is often the case that alternate sub-source-level propositions relating to DNA evidence differ as to whether a male person of interest is the source of DNA in a crime stain or whether the source is one of their close relatives, since both may share the same Y STR profile [3].

Rapidly mutating Y-chromosomal short tandem repeats (RM Y STRs), as found in assays like Yfiler™ Plus (ThermoFisher Scientific, Waltham, MA, USA) and PowePlex^®^ Y23 System (Promega Corporation, Madison, WI, USA), have mutation rates ≥ 10^−2^/locus/generation, in comparison to the standard Y STRs with mutation rates of 10^−3^/locus/generation or lower [4]. RM Y STRs have effectively differentiated close and distant male relatives, where the standard Y STRs have not [5]. Furthermore, their higher diversity has helped to completely differentiate unrelated men [6]. These characteristics make RM Y STRs useful when autosomal STRs are uninformative, especially in crimes involving multiple male perpetrators or in mixed male/female DNA obtained from sexual assault cases [7]. Several studies have confirmed their high mutation rates in haplotype comparisons of populations, genealogies and father–son pairs from Pakistan [8,9,10], Italy [11], Serbia [12], China [13] and Turkey [14]. Using 13 well-established RM Y STRs, Adnan et al. [8] were able to differentiate 27%, 46%, 54% and 62% s of males separated by one, two, three and four generations, respectively. Ralf et al. [15] identified a further 12 RM Y STRs and differentiated 27%, 47% and 61% of males separated by one, two and three generations, respectively. These have been combined into a forensic multiplex assay called RMplex consisting of 26 RM Y STRs and four other fast-mutating Y STRs (FM Y STRs) [3]. Differentiation amongst male relatives can be improved by carefully ascertained additional markers. Hence, there is a need to identify additional markers with high mutation rates in different populations. This could lead to near-complete individualization, particularly in closely related men.

The Pakistani population is endogamous, as 40% of marriages are among first cousins “available online: http://www.pbscensus.gov.pk (accessed on 6 September 2021)”. Such trends elicit a challenge for the differentiation of related males from Pakistan. The discrimination capacity of Y STR markers can be improved either by increasing the number of markers or by selecting markers with high mutation rates. We have employed both strategies and instigated a multiplex for male differentiation in forensic cases involving males with shared ancestry. Here, we present the developmental validation of a RM Y STR multiplex comprising 12 known core loci [16] and 7 additional markers (DYF393S1, DYS464, DYS389 (II), DYS442, DYS635, DYS385 and DYS549). We provide empirical evidence for the differentiation between 536 pairs of male relatives from the endogamous population of Pakistan, ranging from 1 to 7 meiosis as evidence for the paternal lineage resolution ability of 19 RM-YSTRs.

## 2. Materials and Methods

### 2.1. Selection of RM Y-STRs

A total of 19 loci comprising 12 core RM-YSTRs: DYF399S1, DYF387S1, DYF404S1, DYS526 (b), DYS449, DYS518, DYS547, DYS570, DYS576, DYS612, DYS626 and DYS627, along with 7 additional markers: DYS442, DYS635, DYS464, DYS389 (II), DYF393S1, DYS385 and DYS549, were chosen based on their high mutation rates, as published in other populations [1,15,16,17]. All sets of markers chosen in this study, along with their primer sequences and their fragment sizes, are given in Supplementary Table S1. Reference sequences for the primer design were accessed from the NCBI “available online: https://www.ncbi.nlm.nih.gov/ (accessed on 4 January 2019)” and all primer sequences were designed using web-based software Primer3 v4.0, “available online: http://bioinfo.ut.ee/primer3-0.4.0/ (accessed on 4 January 2019)”. Forward primer sequences were labeled with a florescent dye compatible with the matrix standards, and all primers were synthesized by ThermoFisher Scientific.

### 2.2. Sample Collection and DNA Isolation

Buccal swabs of 302 healthy male individuals (97 male rooted pedigrees of 1–4 generations consisting of 536 pairs of male relatives) were collected, with informed consent from the Bahawalpur District of Pakistan (Supplementary Table S2). The pedigrees were consistent, with the relations confirmed through the National Database and Registration Authority (NADRA) and the donors’ Computerized Identity Cards. The study was approved by the Ethical Review Board Committee of the Centre of Excellence in Molecular Biology, University of the Punjab Lahore (Reg: CEMB/AO/076) and was in accordance with the ethical declaration of the World Medical Association Helsinki Declaration (Fortaleza, Brazil, October 2013). Genomic DNA was isolated using organic DNA extraction [18] and was quantified using the Quantifiler™ Human DNA Quantification Kit (ThermoFisher Scientific) on a 7500 SDS Real time PCR System, according to the manufacturer’s recommended protocols. High concentrations of DNA were diluted to a final concentration of 1 ng/µL for optimum amplification.

### 2.3. PCR Multiplexing

Co-amplification of the 19 RM Y STR markers was carried out in a 5-dye florescence-based multiplex assay, ensuring marker balance and no overlapping allelic size ranges (Supplementary Figure S1). Polymerase chain reaction (PCR) was performed in a reaction volume of 8 µL in a Veriti^TM^ thermocycler (Applied Biosystems, Foster City, CA, USA) with the following thermocycler conditions: initial denaturation for 3 min at 95 °C; 30 cycles of 95 °C for 45 s, annealing at 60 °C for 1 min and extension at 72 °C for 1 min; a final extension at 60 °C for 10 min and a hold at 4 °C indefinitely. PCR products were scanned with GeneScan™ 500 LIZ^®^ size standard (Applied Biosystems) and Hi-Di formamide and genotyped on a ABI 3130xl genetic analyzer (Applied Biosystems, Foster City, CA, USA) using the manufacturer’s recommended protocols. Genotyped data were analyzed using GeneMapper ID software, and allele numbers were designated following the guidelines of the International Society of Forensic Genetics (ISFG) [19] and size-based comparisons with control DNA 9948 [9].

### 2.4. Validation Studies

Validation studies were performed using male control DNA, including 9948 for precision and allelic designation and 2800 M for the sensitivity, stability and mixture analysis (all provided by Promega Corporation). For sensitivity testing, serial dilutions of 2800 M (2.0 ng, 1.0 ng, 0.5 ng, 0.25 ng, 0.125 ng and 0.05 ng template amounts) were amplified in triplicate where DNA was diluted using autoclaved nuclease-free water. For stability testing, humic acid (Sigma, St. Louis, MO, USA) was added to the PCRs at concentrations of 25 ng/µL, 75 ng/µL, 85 ng/µL, 100 ng/µL, 125 ng/µL, 175 ng/µL and 225 ng/µL in triplicate and hematin (Sigma, St. Louis, MO, USA) at concentrations of 50 µM, 65 µM, 85 µM, 100 µM, 125 µM, 150 µM, 250 µM, 500 µM and 1000 µM in triplicate. Precision studies were performed in 10 genotyping runs of 9948 standard DNA by calculating the standard deviations for the fragment size variations at each STR locus. The mixture studies were evaluated by preparing (a) male/female mixtures; keeping the female DNA (9947 A) consistent (300 ng) and male DNA serially diluted (2.0 ng, 1.0 ng, 0.5 ng, 0.25 ng, 0.1 ng and 0.05 ng); (b) a mixture of three males at equal concentrations (2800 M and population samples M100 and M103) and (c) various ratios of male/male DNA (2800 M and population sample M103), including 1:1, 3:1, 6:1, 9:1 and 18:1.

### 2.5. Statistical Analysis

PowerMarker software v3.25 [20] was used for estimation of the haplotype frequency, allelic diversity and Polymorphism Information Content (PIC), while the haplotype diversity (HD) was calculated according to:

(1) $$\mathrm{HD}=\frac{n}{n-1}\left(1-\overset{n}{\underset{i=1}{\sum }}x_{i}^{2}\right)$$

where *x_i_* is the (relative) haplotype frequency of the *i*th haplotype, and *n* is the total number of haplotypes [21]. The mutation rate in father–son pairs was calculated as the number of mutations observed at one locus divided by the total number of pairs tested. Binomial standard deviation was used to compute the 95% confidence interval of the mutation rates at each locus [22]. Differentiation for each male relative pair was calculated as the number of pairs separated by at least one mutation divided by the total number of pairs for any particular number of meiosis (1–7).

## 3. Results and Discussion

**Figure 1:**
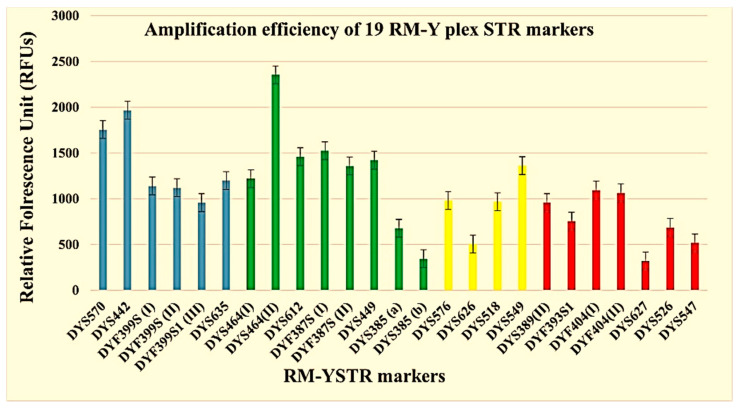
The peak height intensities of each amplified RM Y STR marker based on the 10 genotyping runs of the 9948 control DNA.

Optimization of the multiplex was achieved by varying the primer concentrations (0.31 µM–0.65 µM) in a final reaction volume of 8 µL (Supplementary Figure S2). Sensitivity studies were performed to determine the lowest amount of DNA required to generate a complete DNA profile. Serial dilutions of 2800 M (2.0 ng, 1.0 ng, 0.5 ng, 0.25 ng, 0.125 ng and 0.05 ng) revealed a gradual decrease in the peak intensity (RFU) with a decrease in the concentration, as shown in Supplementary Figure S3. Full profiles were successfully obtained down to 0.125 ng; however, further decreases in the concentration lead to allele dropout for DYS627 and DYS547, and the peak heights for DYS526b and DYS385 were reduced to <100 RFU. Amplification efficiency studies based on ten runs of 9948 standard DNA showed consistent results, as illustrated in Figure 1.

To test the performance of the multiplex in the presence of inhibitors, stability studies were carried out with two inhibition models: hematin and humic acid. The DNA template input amount was 0.5 ng, and the allele peak height threshold was set at 100 RFU. A total of 27 alleles (complete genotype) was obtained at 50 µM, 65 µM, 85 µM, 100 µM and 125 µM concentrations of hematin and at 25 ng, 75 ng, 85 ng, 100 ng and 125 ng concentrations of humic acid. At a concentration of 150 µM of hematin, 19 alleles were ≥100 RFU, while DYS626 dropped to 60 RFU. At a concentration of 250 µM, eight alleles were ≥100 RFU, while three alleles of DYF399S1 were ≤100 RFU, and 13 loci (16 alleles) were completely dropped out. For humic acid at a concentration of 175 ng, 21 alleles were ≥100 RFU, while 6 alleles were entirely dropped out. At a concentration of 225 ng, 18 alleles were ≥100 RFU, and 9 alleles were dropped out completely.

Mixture studies were employed to test the reliability of the multiplex for mixed stains. Firstly, a high but constant female DNA concentration (300 ng) was combined with lower male DNA concentrations in a dilution series (mixture a). Full Y STR profiles were obtained down to 0.05 ng regardless of the female DNA. For the mixture of three males at equal concentrations (mixture b), all expected alleles from the three males were present. For mixtures of two males in various ratios (mixture c), all expected alleles from both males were present at all ratios except 18:1, for which 93% of the expected alleles were present.

### 3.1. Forensic Parameters

Each male-rooted pedigree (*n* = 97) was defined by at least one unique haplotype and therefore achieved 100% discrimination capacity (DC) with HD = 0.991512. The global HD of RM Y STRs has been previously reported by Ballantyne et al. in 2014 as 0.9999985, while, from Pakistan, it has been reported to be 0.9921 in the Sindhi, Brahui and Punjabi regions [7] and 0.993 in the Arain ethnic group [9]. Elsewhere, HD has been found to be 1.00 for South Koreans and Portuguese, 0.9999 for Italians and 0.997 for the UAE [8,23,24,25,26]. RM Y STRs, as for any lineage markers, have lower HD values and a smaller proportion of unique haplotypes in endogamous populations [7]. Among other forensic parameters, the polymorphism information content (PIC) was in the range of 0.511 (DYS549)–0.814 (DYF399S1), and the allelic diversity was in the range of 0.498 (DYS464)–0.814 (DYF399S1).

### 3.2. Mutation Rate Assessment

Mutation rates were calculated for the 19 RM Y STRs based upon 162 father–son pairs selected from 97 pedigrees. The highest mutation rate was observed at DYF399S1 (1.79 × 10^−1^), and the lowest mutation rate was at DYF393S1 and DYS442 (6.17 × 10^−3^). However, no mutations were seen at DYS385 (ab) and DYS549, while the average mutation rate over all the loci was 2.92 × 10^−2^, as shown in Table 1. The mutation rates observed in this study are higher than the mutation rates reported previously from Pakistan [8,9], as compared in Supplementary Table S4.

### 3.3. Differentiation of Male Paternal Relatives

Among the 536 pairs of male relatives, 354 pairs involved at least one mutation at a locus. The pairs were classified as those that were separated by one meiosis (father/son), two meiosis (brothers and grandfather/grandson), three meiosis (great-grandfather/grandson or uncle/nephew), four meiosis (first cousins and great-uncle/nephew), five meiosis (first cousin once removed; the term “removed” indicates the number of generations separating the cousins), six meiosis (second cousin) and seven meiosis (second cousin once removed). At least one mutation of the 19 RM Y STR markers was observed in 44%, 69%, 68%, 85%, 0%, 100% and 100% of these cases, respectively, as shown in Table 2.

**Figure 2:**
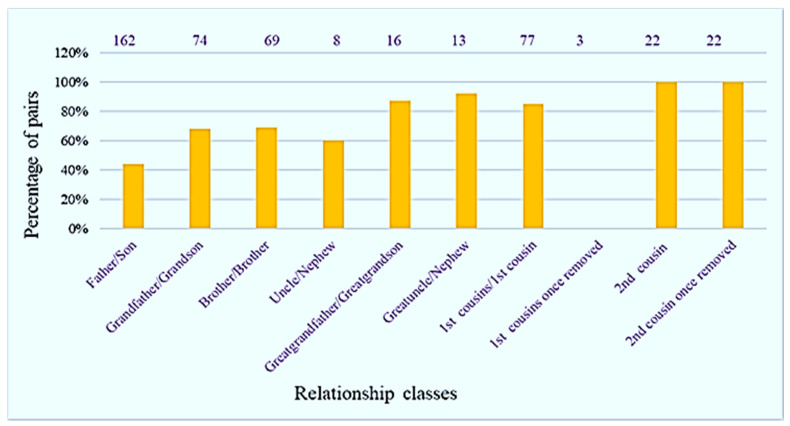
Pairs of male paternal relatives (*n* = 536) separated by one to seven meiosis with at least one mutation. Values above the bars represent the total number of pairs for each relationship class.

The proportions of pairs that could be differentiated therefore increased with the number of meiosis (Figure 2), with five meiosis representing an outlier (no differentiation). There were only three pairs in this category, and the small sample size may be responsible for the anomaly. Ballantyne et al., in 2014 [7], reported that RM Y STRs increased the overall HD in 86% of examined populations and improved the ability to differentiate between male paternal relatives by 23% in comparison to Yfiler™ (ThermoFisher Scientific). Rakha et al. [28] also found that RM Y STRs differentiated between paternal male relatives for 39% of cases, compared with only 9% and 7% for Yfiler and PowerPlex Y23, respectively. In a previous study [9], we observed 68 mutations in at least one of 12 RM Y STRs in 35 pairs of male relatives with a mutation rate of 7.14 × 10^−2^/locus/generation. According to Table 2, the mutation rates in this study were all higher than previous studies [8,16], except for five meiosis. Our mutation rates (and potential to differentiate between male paternal relatives) were consistent with those of Ralf et al. [15] from 26 Y STRs after various numbers of meiosis.

In 2022, Franz and collaborators [27] were able to differentiate between 42% of male paternal relatives in 499 European father–son pairs using a 30 YSTR multiplex called RMplex, while Yfiler™ Plus was only able to differentiate between 14% of male paternal relatives in 530 pairs. RMplex and Yfiler™ Plus combined were able to differentiate between 48% of male paternal relatives, which was higher than observed using RMplex alone. For brothers, RMplex was able to differentiate between 62% of male paternal relatives, compared to 33% with Yfiler Plus [28]. No data were available for male paternal relatives separated by three or more meiosis. Our multiplex performed at least as well as RMplex, even though it has less STRs. However, most of the markers included in RMplex are not yet characterized in Pakistani populations.

### 3.4. Differentiation Potential of Individual RM Y STR Loci

The differentiation potential of 19 RM Y STRs was calculated in 97 pedigrees at all numbers of meiosis. The highest differentiation potential was observed at DYF399S1, DYS626 and DYS547 by the total number of mutations (197, 55 and 51), respectively (Table 3), whereas the highest number of mutations was observed among first cousins, followed by father–son, second cousins and second cousin once removed, at DYF399S1. While, at 19 loci, 96 mutations were observed in the first meiosis, 100 mutations in pairs separated by two meiosis, 96 mutations at three meiosis, 128 mutations at four meiosis, no differentiations after five meiosis, 36 mutations at six meiosis and 35 mutations at seven meiosis, as shown in Table 3. Mutations at multiple RM Y STR loci were observed for some pairs of male paternal relatives: from two to three mutations after one meiosis to five mutations after seven meiosis. A maximum of four mutations was most commonly observed at other numbers of meiosis in the dataset. One constraint linked to male relatives from pedigrees is that one mutation is counted several times, depending upon the number of relations within a pedigree or the position of pedigree of the members presenting the mutation (Supplementary Figure S5). This might lead to an overestimation of the differentiation rates among individuals separated by more than one meiosis. This limitation can be overcome by selecting all the related pairs from independent pedigrees; it would require a large number of pedigrees.

### 3.5. Multi Copy Variations

The current data comprises 196 copy number variations at DYF387S1, DYS464 and DYF404S1 (Supplementary Table S3). Hexaplicate alleles were observed in three families (four times) at DYS464 and DYF387S (Supplementary Figure S4A). Similarly, 10 families showed pentaplicate alleles at DYS464 and DYF387S1 (31 times), while quadruplicate alleles were observed in 42 families (105 times) and triplicate alleles were examined in 13 families at DYF387S1 and one family at DYF404S1 (54 times). Though the occurrences of triplicate alleles at DYF387S1 and DYF404S1 (Supplementary Figure S4B) have been seldom reported [29,30,31], no pentaplicate or hexaplicate alleles at DYF387S1 and DYF464 have been previously reported. It might be caused by duplication events or otherwise nonallelic homologous recombinations. It is believed that the diversity observed at DYF387S1 is shaped by nonallelic gene conversions [32]. In spite of the fact that copy number variations at Y-STRs can be applied in the interpretation of mixed DNA sources, certainly, the reason for the variant formations at each locus need further investigation [3].

**Figure 3:**
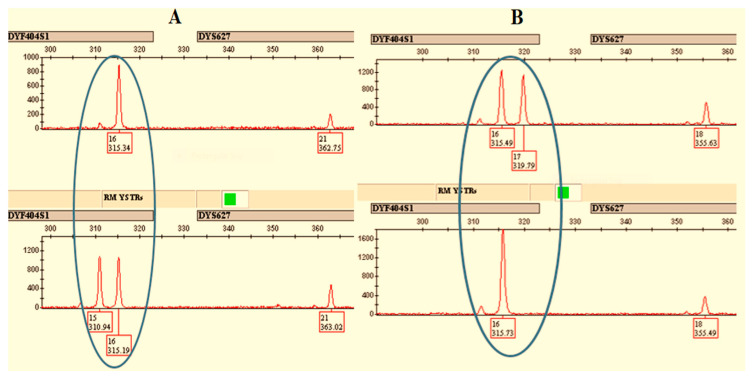
Electropherograms showing mutations observed at various RM Y STR loci for (**A**) a father–son pair and (**B**) a brother–brother pair, where the mutation occurs at the duplicated locus DYF404S1. Circles indicate probable mutation events.

Genotypes from two paternally related males for DYF404 that typically have two copies on the Y chromosome (CNV = 2) are shown in Figure 3A. In the upper electropherogram, the genotype appears to be 16, while, in the bottom electropherogram, the genotype is 15/16. If the possibility of locus deletion is ignored, this suggests that the true genotype for the father in the upper electropherogram is 16/16 instead of 16, and a mutation occurred for one copy, such that a 16 allele lost a repeat unit to become 15 in the son. In Figure 3B, the upper electropherogram represents genotype 16/17, while the bottom electropherogram appears to show genotype 16. Once again, a valid interpretation is that the true genotype for the bottom electropherogram is 16/16 instead of 16, if the possibility of locus duplication during meiosis is ignored. This suggests that a mutation occurred such that one of the brothers had a mutation, either 16→17 (upper electropherogram) or 17→16 (lower electropherogram). The possibility of a locus duplication (upper electropherogram) or deletion (lower electropherogram) during meiosis cannot be ignored, however. It is possible that sequencing could distinguish between CNVs with the same alleles. Allele peak heights could also be used to indicate CNVs, but the peak height differences might be the result of PCR stochastic effects. As such, a conservative approach is to not take the RFU into account, but this reduces the ability to differentiate between male relatives. The most common mutational events observed are described in Table 4.

## 4. Conclusions

The newly designed RM Y STR multiplex presented here is a useful complement to conventional Y STR kits for the lineage identification and individualization of paternally related males. Validation studies have revealed that it is a robust, efficient and sensitive system for RM Y STR genotyping. A total of 97 endogamous pedigrees were analyzed with a newly designed multiplex for differentiating related males. This 19 RM Y STR panel shows a high level of differentiation between one and seven meiosis. An overall differentiation rate of 66% was obtained for paternally related male pairs separated by one to seven meiosis in 97 endogamous pedigrees. This is far in excess of the differentiation rates obtainable from commercially available multiplexes (PowerPlex Y23 and Yfiler Plus) and at least as effective as other multiplexes with more STRs. Triplicate, quadruplicate, pentaplicate and hexaplicate alleles were observed at two loci (DYS464 and DYF387S1). Further studies should be conducted for more data in major global populations to gain insights into the haplogroup effects on the mutation rate estimates at these loci. Moreover, carefully ascertained additional Y STR markers and population-specific mutation rates can improve the differentiation power among male relatives as a step towards the complete individualization of paternally related males. This study contributed data to expanding the global databases of RM Y STRs.

## Figures and Tables

**Table 1:** Mutation rates observed at 19 loci in father–son pairs (*n* = 162).

Locus	Mutations Observed	Mutation Rate (95% Confidence Interval)
DYF399S1	29	1.79 × 10^−1^ (1.23 × 10^−1^–2.46 × 10^−1^)
DYF387S1	2	1.23 × 10^−2^ (1.49 × 10^−3^–4.38 × 10^−2^)
DYF404S1	2	1.23 × 10^−2^ (1.49 × 10^−3^–4.38 × 10^−2^)
DYS526 (b)	3	1.85 × 10^−2^ (3.83 × 10^−3^–5.31 × 10^−2^)
DYS389 (II)	3	1.85 × 10^−2^ (3.83 × 10^−3^–5.31 × 10^−2^)
DYF393S1	1	6.17 × 10^−3^ (1.56 × 10^−4^–3.39 × 10^−2^)
DYS449	3	1.85 × 10^−2^ (3.83 × 10^−3^–5.31 × 10^−2^)
DYS464	7	4.32 × 10^−2^ (1.75 × 10^−2^–8.70× 10^−2^)
DYS518	3	1.85 × 10^−2^ (3.83 × 10^−3^–5.31 × 10^−2^)
DYS442	1	6.17 × 10^−3^ (1.56 × 10^−4^–3.39 × 10^−2^)
DYS547	8	4.93 × 10^−2^ (2.15 × 10^−2^–9.49 × 10^−2^)
DYS570	8	4.93 × 10^−2^ (2.15 × 10^−2^–9.49 × 10^−2^)
DYS576	7	4.32 × 10^−2^ (1.75 × 10^−2^–8.70× 10^−2^)
DYS612	2	1.23 × 10^−2^ (1.49 × 10^−3^–4.38 × 10^−2^)
DYS626	13	8.02 × 10^−2^ (4.34 × 10^−2^–1.33 × 10^−1^)
DYS627	2	1.23 × 10^−2^ (1.49 × 10^−3^–4.38 × 10^−2^)
DYS635	2	1.23 × 10^−2^ (1.49 × 10^−3^–4.38 × 10^−2^)
DYS385	0	0.000 (0–2.25 × 10^−2^)
DYS549	0	0.000 (0–2.25 × 10^−2^)
Total	96	3.11 × 10^−2^ (2.53 × 10^−2^–3.79 × 10^−2^)

**Table 2:** Comparison of the mutations observed at 19 RM Y STR and previously observed in 13 RM-YSTR loci [8,16,27].

Relationship Pairs	Meiosis	Mutations in 19 RM-Y STR Loci			Mutations Previously Observed in 13 RM Y STR Loci	
		Total Number of Pairs	Pairs with at Least One Mutation	Proportion with at Least One Mutation	Total Number of Pairs	Proportion with at Least One Mutation
Father/Son	1	162	72	44%	2378, 428, 499	29%, 24%, 42%
Grandfather/Grandson	2	74	51	68%	801, 480	44%, 44%
Brother/Brother	2	69	48	69%		
Uncle/Nephew	3	78	47	60%	507, 308	53%, 54%
Greatgrandfather–son	3	16	14	87%		
Greatuncle/Nephew	4	13	12	92%	533, 277	63% 60%
1st cousins	4	77	66	85%		
1st cousins once removed	5	3	0	0%	231, 43	77% 70%
2nd cousin	6	22	22	100%	76, 32	74%, 75%
2nd cousin once removed	7	22	22	100%	14, 0	28%
Overall		536	354	66%	6108	39%

**Table 3:** Number of mutations identified at each RM Y STR in the studied pairs of male paternal relatives.

Locus	Father–Son (*n* = 162)	Grandfather–Grandson (*n* = 74)	Brother Pairs (*n* = 69)	Uncle–Nephew (*n* = 78)	Great Grandfather–Great Grandson (*n* = 16)	Great Uncle–Nephew (*n* = 13)	First Cousin (*n* = 77)	First Cousin Once Removed (*n* = 3)	Second Cousin (*n* = 22)	Second Cousin Once Removed (*n* = 22)	Totals
DYF399S1	29	16	12	25	9	13	42	0	26	25	197
DYF387S1	2	2	0	0	0	0	0	0	0	0	4
DYF404S1	2	3	2	1	12	0	0	0	0	0	20
DYS526 (b)	3	3	2	4	2	0	20	0	0	0	34
DYS389(II)	3	1	0	1	0	0	1	0	0	0	6
DYF393S1	1	0	4	1	0	0	0	0	0	0	6
DYS449	3	2	2	3	0	0	0	0	0	0	10
DYS464	7	2	1	2	0	3	1	0	10	10	37
DYS518	3	3	1	2	0	0	1	0	0	0	10
DYS442	1	1	0	1	0	0	1	0	0	0	4
DYS547	8	6	6	1	3	0	27	0	0	0	51
DYS570	8	0	1	3	0	0	3	0	0	0	15
DYS576	7	2	2	4	0	0	3	0	0	0	18
DYS612	2	6	4	2	0	0	0	0	0	0	14
DYS626	13	3	8	8	11	0	12	0	0	0	55
DYS627	2	1	0	1	0	1	0	0	0	0	5
DYS635	2	2	2	0	0	0	0	0	0	0	6
DYS385	0	0	0	0	0	0	0	0	0	0	0
DYS549	0	0	0	0	0	0	0	0	0	0	0
Totals	96	53	47	60	36	17	111	0	36	35	492

**Table 4:** Mutation events observed at various RM Y STR loci.

STR Loci	Mutations Events	Steps of Mutations						
		1	2	3	4	5	6	7
DYF399S1	21→20, 24→25, 23→22.3, 22.3→23.1, 23.2→22.3, 27→26, 22.3→22, 21→21.1, 23→22.3, 27→26, 24→23.3, 21→22, 25→23.3, 25→24, 28→27, 25→26, 26.3→27.3, 25→23.3.	194	3	0	0	0	0	0
DYF387S1	39→40, 38→null	4	0	0	0	0	0	0
DYF404S1	null→15, 14→13, 13→14, 17→null	20	0	0	0	0	0	0
DYS526 (b)	36→35, 39→40, 40→39.2, 39→39.2,	34	0	0	0	0	0	0
DYS389 (II)	32→33, 33→32, 30→28, 28→30	6	2	0	0	0	0	0
DYF393S1	28→27, 30→28, 30→28	3	3	0	0	0	0	0
DYS449	32→31, 27→28, 33→32, 32→31	10	0	0	0	0	0	0
DYS464	12→11, 12→13, 15→15.2, 15→null, 17→null	37	0	0	0	0	0	0
DYS518	39→40, 40→39, 41.3→39, 41→42	10	0	0	0	0	0	0
DYS442	13→12	4	0	0	0	0	0	0
DYS547	50→49, 47→46, 49→48, 48→49, 48→43, 43→48, 50→48, 48→50	46	3	0	0	2	0	0
DYS570	19→18, 17→16, 18→17, 18→19, 18→17	15	0	0	0	0	0	0
DYS576	18→16, 19→18, 19→18.3, 18→22	13	2	0	3	0	0	0
DYS612	37→36, 39→40, 37→36, 37→38, 36→34	11	3	0	0	0	0	0
DYS626	31→27, 26→33, 27→26, 27→32, 26→32, 32→27, 31→30, 28→28.2, 33→32	7	0	0	2	38	2	6
DYS627	23→24, 22→18, 22→21, 18→22, 18→21,	2	0	1	2	0	0	0
DYS635	24→23, 24→25	6	0	0	0	0	0	0

## Data Availability

Data are contained within the article or Supplementary Materials.

## Supplementary Materials

The following supporting information can be downloaded at: https://www.mdpi.com/article/10.3390/genes13060946/s1, Figure S1: Schematic representation of florescent dye colors with marker product allelic size ranges for the 19 RM Y STR markers incorporated in the multiplex; Figure S2: Electropherogram of control DNA 9948 analyzed with the 19 RM Y STR multiplex at varying primer concentration (0.31 µM–0.65 µM); Figure S3: Serial dilutions of 2800M control DNA display a gradual decrease in RFU with decrease in concentration; Figure S4: (A) Hexaplicate alleles observed at DYS464 and DYF387S1 (B) Triplicate allele observed at DYF404S1; Figure S5: (A) Paternal lineages of family ID 53 and (B) family ID 57 has been illustrated as representing mutation at one or more loci with red symbols while subtext showing the alleles; Table S1: RM Y STR primer sequences, repeat structures and allelic size ranges; Table S2: Pedigrees and sample information along with no. of generations, meiosis, pairwise meiosis and their mutations events; Table S3: Copy number variants observed at duplicated loci; Table S4: The mutation rate found in present and previous studies for 19 Y STRs.
