# Supplementary material for: Empirical Evidence on Enhanced Mutation Rates of 19 RM-YSTRs for Differentiating Paternal Lineages
Source: Genes (Basel). 2022 May 26;13(6):946. doi: 10.3390/genes13060946 (PMC9222627; doi:10.3390/genes13060946)
Supplement: Supplementary file 1 [file genes-13-00946-s001.zip › genes-1701635-supplementary.pdf]

Supplementary Table S1: RM Y STR primer sequences, repeat structures and allelic size ranges.					
	**Marker name**	**Repeat motif**	**Primer Sequence**	**Allelic size range (bp)**	**Reference**
1	DYS570	(TTTC)$_{14–24}$	Forward- FAM-AAATCCTGGCTGTGTCCTC<br>Reverse- TGGCAACCTAAGCTGAAATG	114-160	[9]
2	DYS442	**(GATA)** $_{9-16}$(GACA)$_{3}$	Forward-FAM-CAATCTCATCCAAGCCACT<br>Reverse- AATTGCAAAATCACGGAACC	190-216	This study
3	DYF399S1	(GAAA)3N $_{7–8}$(GAAA)$_{10–23}$	Forward-FAMGAACCAAGGGAAATGTGGAA<br>Reverse: CTGGACAGCTTAGGATTGGAC	246-302	[9]
4	DYS635	(TCTA)$_{4}$(TGTA)$_{2}$(TCTA)$_{2}$(TGTA)$_{2}$ (TCTA)$_{2}$ **(TATG)** $_{0-2}$ **(TCTA)** $_{4-17}$	Forward-FAM-GCAGCAAAATTCACAGTTGG<br>Reverse-GGCTGATTAGTATTCCATGGTG	369-408	This study
5	DYS464	**(CCTT)** $_{9-20}$N$_{46}$(CCTT)$_{3}$N$_{8}$(CCTT)$_{4}$	Forward-VIC- TTACGAGCTTTGGGCTATGC<br>Reverse- AAGGAAGGGAGGGATGGAG	125-174	This study
6	DYS612	(CCT)$_{5}$(CTT)$_{1}$(TCT)$_{4}$(CCT)$_{1}$(TCT)$_{19–31}$	Forward-VIC-CCCCCATGCCAGTAAGAATA<br>Reverse: AAAAGGGAACTGAGGGAAGG	196-226	[9]
7	DYF387S1	(AAAG)$_{3}$(GTAG)$_{1}$(GAAG)$_{4}$N$_{16}$ (GAAG)9(AAAG)$_{13}$	Forward-VIC-GGGTGACAGAGCTAGATTCC<br>Reverse: TGGTGCCACAGTGTGAGAAG	250-286	[9]
8	DYS449	(TTCT)$_{13–19}$N22(TTCT)$_{3}$N12(TTCT)$_{13–19}$	Forward-VIC-TTTAGGCTAGAGATTCTTGGAGTC<br>Reverse: TCTAGGTTGGACAACAAGAGTAAGAC	317-357	[9]
9	DYS385a<br>DYS385b	(AAGG)$_{4}$N$_{14}$(AAAG)$_{3}$N$_{12}$(AAAG)$_{3}$N$_{29}$ **(AAGG)** $_{6–7}$ **(GAAA)** $_{7-23}$<br>(AAGG)$_{4}$N$_{14}$(AAAG)$_{3}$N$_{12}$(AAAG)$_{3}$N$_{29}$<br>**(AAGG)** $_{6-7}$ **(GAAA)** $_{7-23}$	Forward-VICCAGCATGGGTGACAGAGCTA<br>Reverse- ATGCCCGCTACTCAATGTTC	395-442	This study
10	DYS576	(AAAG)$_{13–22}$	Forward-NEDTTGGGCTGAGGAGTTCAATC<br>Reverse-TTCCTGGAGATGAAGGAGGA	163-207	[9]
11	DYS626	(GAAA)$_{14–23}$N$_{24}$(GAAA)$_{3}$N$_{6}$(GAAA)$_{5}$(AAA)$_{1}$ (GAAA)$_{2–3}$(GAAG)$_{1}$(GAAA)	Forward-NED-TGGGTGACAGAGTGCAAGAC<br>Reverse- GAAGAATTTTGGGACATGTTTG	235-267	[9]
12	DYS518	GAAA[2]GGAA[1]**GAAA[15-16]**GGGA[1]**GAAA[4]**GGAA[1]GA[1]**GAAA[11-14]**GGAA[1]GA[1]GGGA[2]AGAA[1]GAAA[1]AGAA[2]**GGAA[3]**	Forward-NEDATCCCAACTACTCATGAGGCT<br>Reverse- CACATCTTCAGCTCTTACCATGG	352-404	This study
13	DYS549	**(GATA)** $_{9-15}$	Forward-NED-CCAATGAACCCCACCACAAG<br>Reverse-TGTGTGCATAGAGGTGTTCAGA	407-423	This study
14	DYS389 II	(TCTG)$_{5}$ **(TCTA)** $_{12}$N$_{28}$(TCTG)$_{3}$(TCTA)$_{10}$	Forward-PET-CTGCATTTTGGTACCCCATA<br>Reverse- TGAGTGGGAGAAATGGATGA	189-229	This study

15	DYF393S1	$(AAG)_4(AA)_1\ \mathbf{(AAG)}_{16\text{-}30}(CAG)_{1\text{-}2}$	Forward-PET-CAGACTGGCAACCAAAAGG<br>Reverse- GATGTCTGCGTGTGTGTGTG	232-273	This study
16	DYF404S1	$(TTTC)_{10\text{–}20}\ N_{42}(TTTC)_3$	Forward-PET-GGCAGGACACATTTAAACAA<br>Reverse- GCCAGGTATTCTGGTTGAGG	290-318	[9]
17	DYS627	$(AGAA)_3N_{16}(AGAG)_3(AAAG)_{12\text{–}24}N_{81}\ (AAGG)_3$	Forward-PET-GCACAGATAATGCCACTGC<br>Reverse-CCATGTGGATAATGAGCAAA	333-377	[9]
18	DYS526b	$(CCCT)_3N_{20}(CTTT)_{11\text{–}17}(CCTT)_{6\text{–}10}N_{113}\ (CCTT)_{10\text{–}17}$	Forward-PET-CAGCAAACACCTTAGCTAATCA<br>Reverse-AGTAAACGTTTGGGTTACTTCG	400-446	[9]
19	DYS547	$(CCTT)_{9\text{–}13}T(CTTC)_{4\text{–}5}N_{56}(TTTC)_{10\text{–}22}N_{10}(CCTT)_4(TCTC)_1(TTTC)_{9\text{–}16}N_{14}(TTTC)_3$	Forward-PET-TGACCTCTAGTTCCAATTCTATCCA<br>Reverse- CAGCCTGAGTGACAGAGCAT	450-485	[9]

Supplementary Table S2: Pedigrees and sample information along with no. of generations, meiosis, pairwise meiosis and their mutations events.

**Pedigree no.**	**No. of Individuals**	**No. of generations**	**Meioses**	**Pairwise Meioses**	**Mutational events RM Y-STRs**
1	4	2	2	6	2
2	3	2	2	3	0
3	2	2	1	1	1
4	3	3	2	4	2
5	2	2	1	1	1
6	2	1	1	1	1
7	2	2	1	1	0
8	2	3	2	1	1
11	2	3	2	1	1
12	2	2	1	1	1
14	2	3	2	1	0
10	3	3	2	4	2
16	3	3	2	4	1
17	2	2	1	1	1
18	3	3	2	4	3
20	2	1	1	1	0
21	2	1	1	1	0
22	2	2	1	1	1
23	2	1	1	1	2
24	2	2	1	1	0
25	3	3	2	4	2
26	3	3	2	4	3
27	3	3	2	4	1

28	2	1	1	1	0
29	3	3	2	4	0
30	2	3	1	1	1
31	2	2	1	1	0
32	2	2	1	1	0
33	2	3	1	1	1
34	2	1	1	1	0
35	3	3	2	4	0
36	2	1	1	1	0
37	2	2	1	1	0
38	2	1	1	1	1
39	5	3	4	20	1
40	2	1	1	1	0
41	2	2	1	1	0
42	2	1	1	1	1
43	2	1	1	1	0
44	5	3	4	20	1
45	2	2	1	1	0
46	2	2	1	1	2
47	4	3	3	6	4
48	3	2	2	4	0
49	2	2	1	1	0
50	2	2	1	1	0
51	2	2	1	1	0
52	2	2	1	1	0
53	4	3	3	10	2
54	5	4	4	22	3
55	6	4	5	32	2
56	2	2	1	1	0
57	8	3	7	72	6
58	17	4	16	276	15
59	9	3	8	90	2
60	2	2	1	1	2
61	4	3	3	10	1
62	16	4	14	335	14
63	4	3	3	10	3
64	4	2	4	12	4
65	2	2	1	1	0
66	2	2	1	1	0
67	4	2	3	9	0
68	2	2	1	1	0

69	2	2	1	1	1
70	4	2	3	9	4
71	4	3	3	10	2
72	3	2	2	4	0
73	2	2	1	1	0
74	2	2	1	1	0
75	2	2	1	1	1
76	4	3	3	10	5
77	4	3	3	10	6
78	5	3	4	18	1
79	5	3	4	18	0
80	6	3	5	30	1
81	2	2	1	1	0
82	2	2	1	1	0
83	3	3	2	6	4
84	2	2	1	1	0
85	3	2	3	4	2
86	3	2	2	4	1
87	2	2	1	1	1
88	3	2	2	4	2
89	2	2	1	1	0
90	4	3	3	10	1
91	2	2	1	1	0
92	3	2	2	4	0
93	2	2	1	1	0
94	2	1	1	1	0
95	2	1	1	1	0
96	2	1	1	1	0
97	4	3	3	10	0
98	4	3	3	10	0
99	2	1	1	1	1
100	2	1	1	1	0
Total	302	NA	209	1188	126

Supplementary Table S3: Copy number variants observed at duplicated loci

Locus	Genotype	CNV type	No. of individuals showed the genotype	9948 Genotype
DYS464	12,13,14,15.2,16	Pentaplicate	3	15,16
DYS464	14,15,16,17,19	Pentaplicate	5	15,16
DYS464	12,14,15,16,20	Pentaplicate	3	15,16
DYS464	14,15,17,18,19,20	Hexaplicate	2	15,16
DYS464	10.3,12,14,15,16	Pentaplicate	2	15,16
DYS464	11,13,15,15.2,16	Pentaplicate	3	15,16
DYS464	13,14,15,15.3,16, 17	Hexaplicate	2	15,16
DYS464	13,14,16,17,19	Pentaplicate	3	15,16
DYS464	12,14,15,16,17	Pentaplicate	9	15,16
DYS464	12,13,14,15,16	Pentaplicate	2	15,16
DYS464	10.2,14,15,16,17	Pentaplicate	1	15,16
DYS464	10.2,14,15.2,16	Quadruplicate	1	15,16
DYF387S1	36,37,39,40	Quadruplicate	13	35,38
DYF387S1	36,37,41,42	Quadruplicate	11	35,38
DYF387S1	37,38,39,40	Quadruplicate	19	35,38
DYF387S1	37,38,39,41,42	Quadruplicate	2	35,38
DYF387S1	37,38,39,40,41	Quadruplicate	2	35,38
DYF387S1	37,38,40,41	Quadruplicate	6	35,38
DYF387S1	37,38,41,42	Quadruplicate	2	35,38
DYF387S1	38,39,42,43	Quadruplicate	2	35,38
DYF387S1	38,40,42,43	Quadruplicate	1	35,38
DYF387S1	36,37,38,39	Quadruplicate	16	35,38
DYF387S1	35,36,38,39	Quadruplicate	4	35,38
DYF387S1	35,36,39,40	Quadruplicate	6	35,38
DYF387S1	37,38,38.1,39	Quadruplicate	2	35,38

DYF387S1	35,36,40,41	Quadruplicate	2	35,38
DYF387S1	37,38,38.2,39.2	Quadruplicate	3	35,38
DYF387S1	36,37,37.2,39,41,42	Hexaplicate	2	35,38
DYF387S1	35,36,37,38	Quadruplicate	6	35,38
DYF387S1	38,39,40,41	Quadruplicate	4	35,38
DYF387S1	34,35,38,39	Quadruplicate	3	35,38
DYF387S1	37,38,39	Triplicate	50	35,38
DYF387S1	36,38,39	Triplicate	1	35,38
DYF387S1	36,37,38	Triplicate	2	35,38
DYF404	13.3,15,16	Triplicate	1	12,14
Total			196	

Supplementary Table S4: The mutation rate found in present and previous studies for 19 Y STRs						
	Faqeeha (Present study)	Neuhuber (2022)	Ralf (2021)- cell line DNA	Adnan 2016	Ballantyne 2010	Ballantyne 2014
DYF399S1	197/536	41/530	2/93 pairs	27/428	139/1794	51/911
DYS449	10/536	11/530	0/93 pairs	2/428	19/1617	11/911
DYS612	14/536	8/530	1/93 pairs	14/428	25/1767	19/911
DYS526b	34/536	7/528	0/93 pairs	6/428	20/1651	12/911
DYS570	15/536	6/530	0/93 pairs	4/428	17/1426	9/911
DYS385	0/536	6/530	0/93 pairs			
DYF387S1	4/536	6/530	1/93 pairs	10/428	28/1804	4/911
DYS576	18/536	5/530	0/93 pairs	6/428	24/1727	9/911
DYF404S1	20/536	3/530	1/93 pairs	5/428	21/1739	12/911
DYS547	51/536	3/529	0/93 pairs	8/428	39/1679	12/911
DYS626	55/536	2/529	1/93 pairs	3/428	20/1689	8/911
DYS627	5/536	2/530	0/93 pairs	3/428	21/1766	19/911
DYS518	10/536	2/530	2/93 pairs	14/428	28/1556	18/911
DYF393S1	6/536	2/530	1/93 pairs			
DYS442	4/536	1/528	0/93 pairs			
DYS389II	6/536	0/530	0/93 pairs			
DYS464	37/536					
DYS635	6/536	0/530			6/1732	
DYS549	0/536					

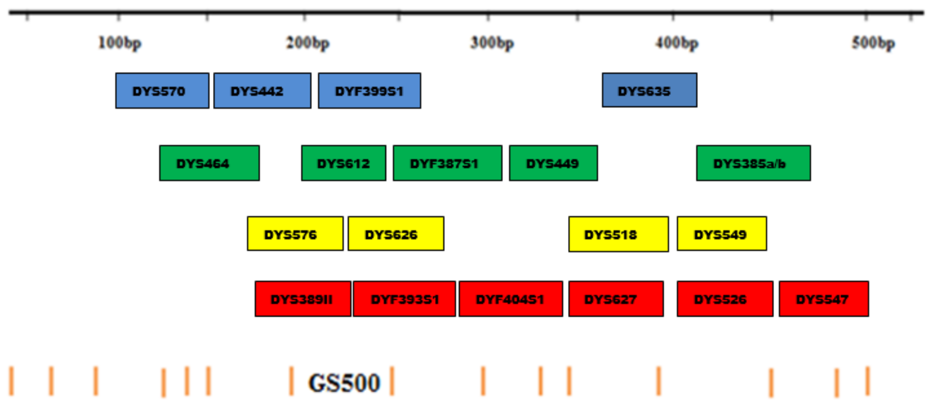

Supplementary Figure S1: Schematic representation of florescent dye colors with marker product allelic size ranges for the 19 RM Y STR markers incorporated in the multiplex.

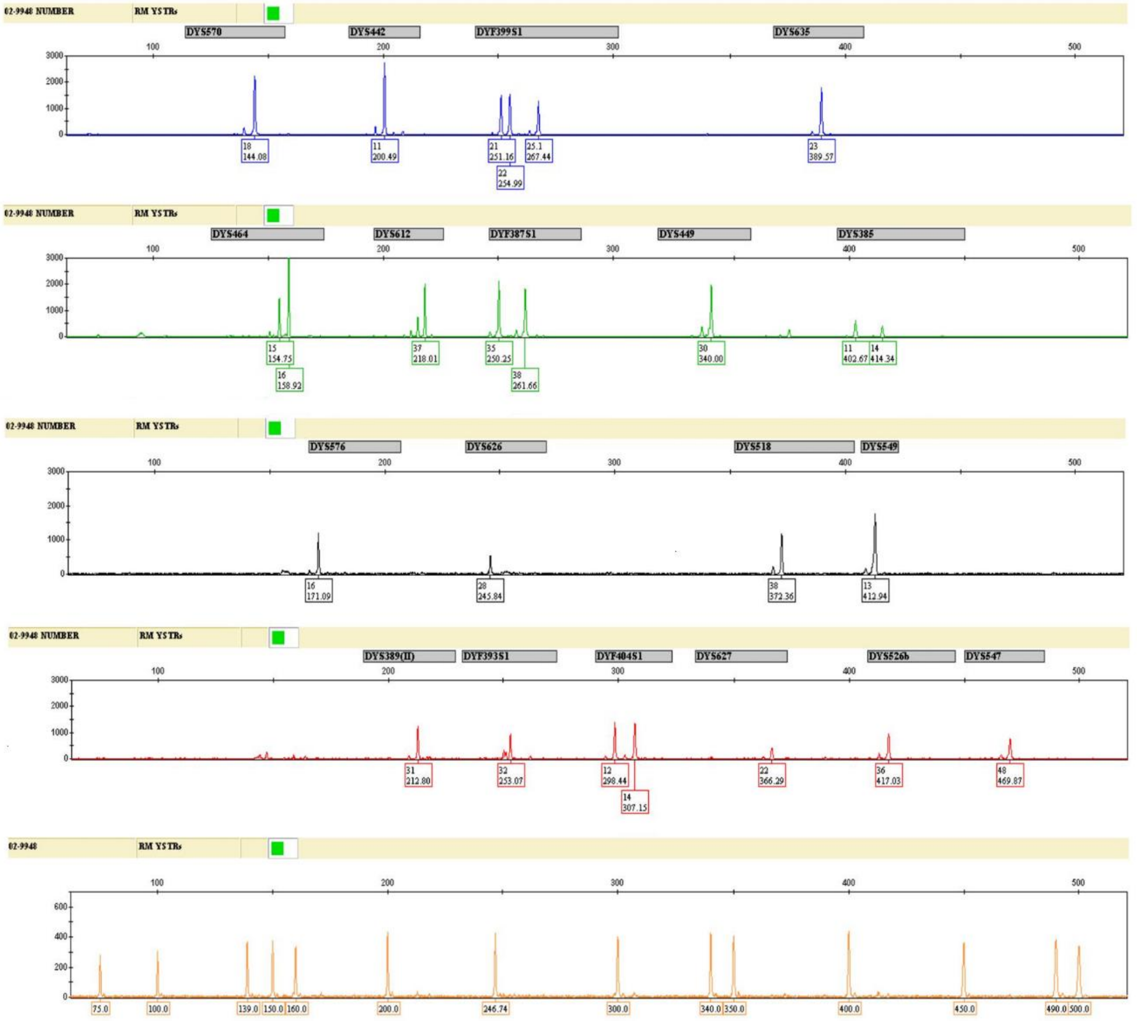

Supplementary Figure S2: Electropherogram of control DNA 9948 analyzed with the 19 RM Y STR multiplex at varying primer concentration (0.31 μM - 0.65 μM).

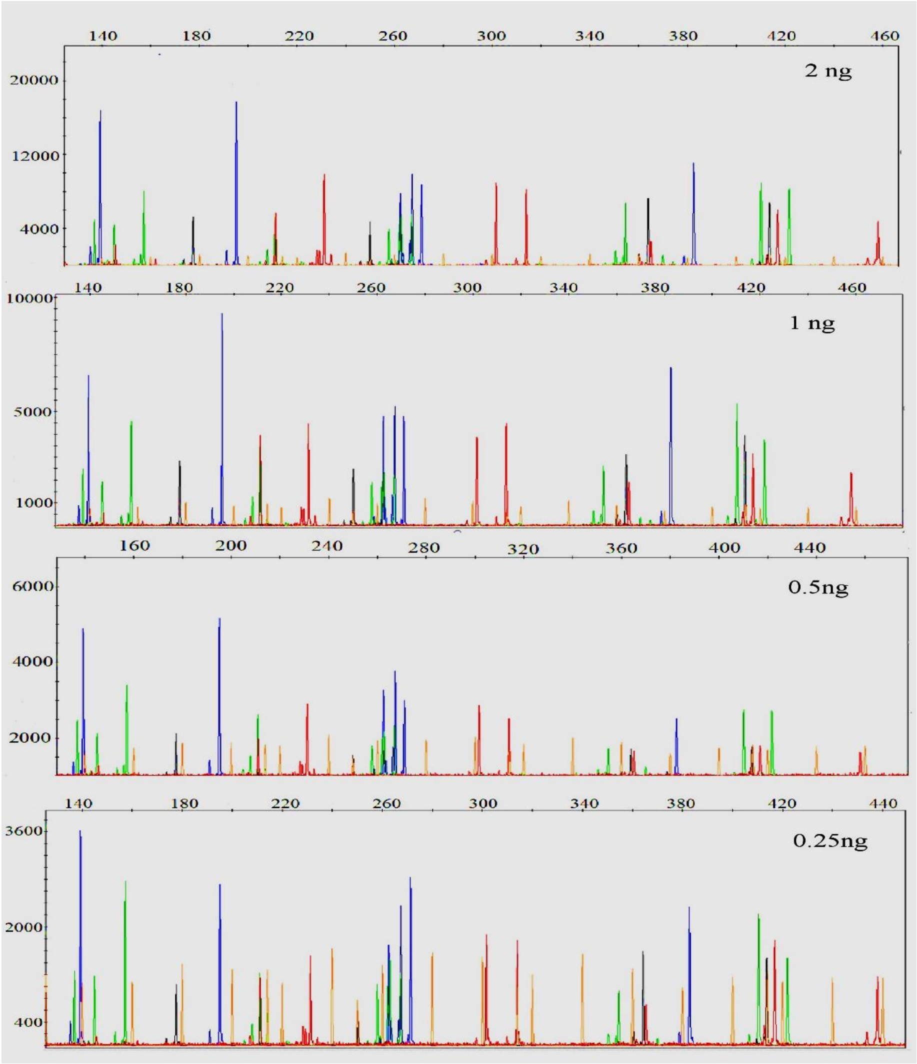

Supplementary Figure S3: Serial dilutions of 2800M control DNA display a gradual decrease in RFU with decrease in concentration.

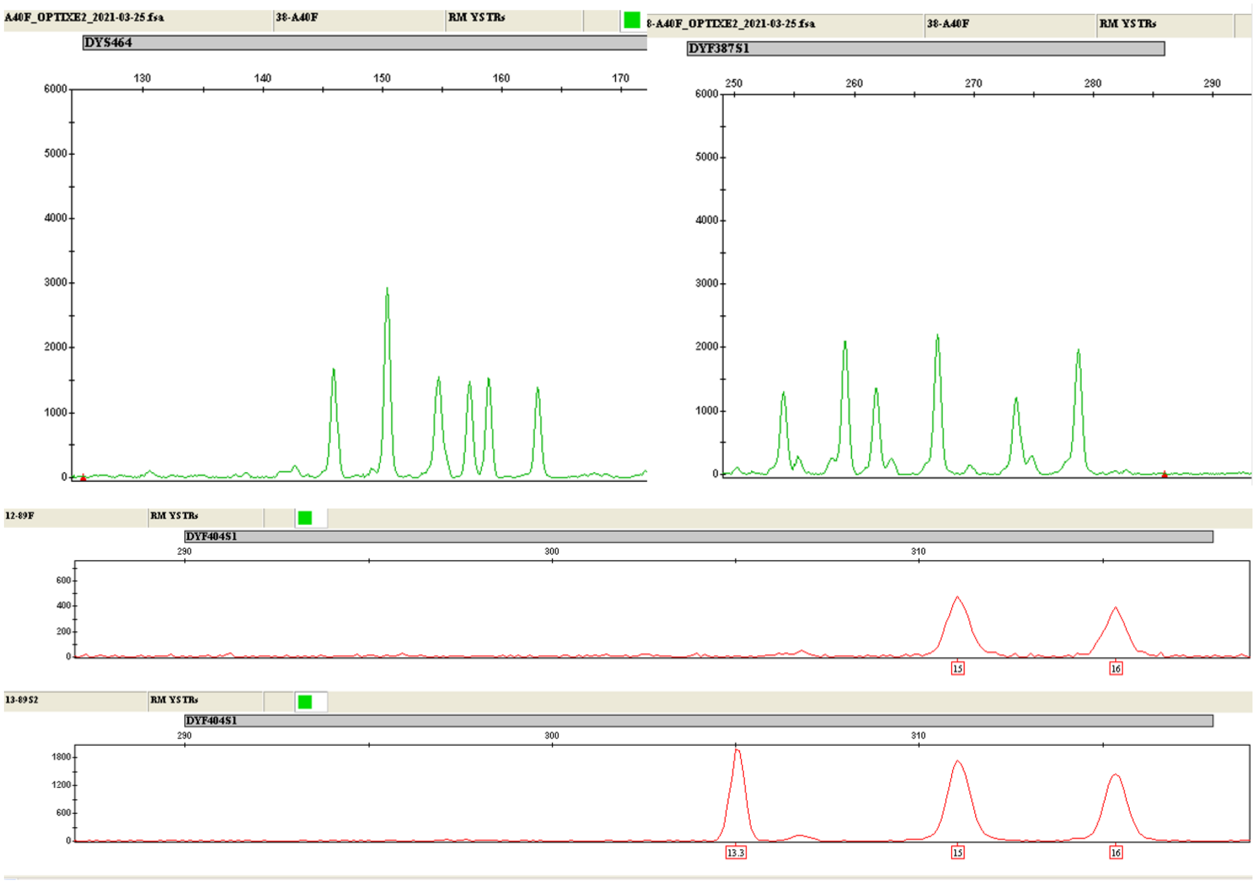

Supplementary Figure S4: (A) Hexaplicate alleles observed at DYS464 and DYF387S1 (B) Triplicate allele observed at DYF404S1

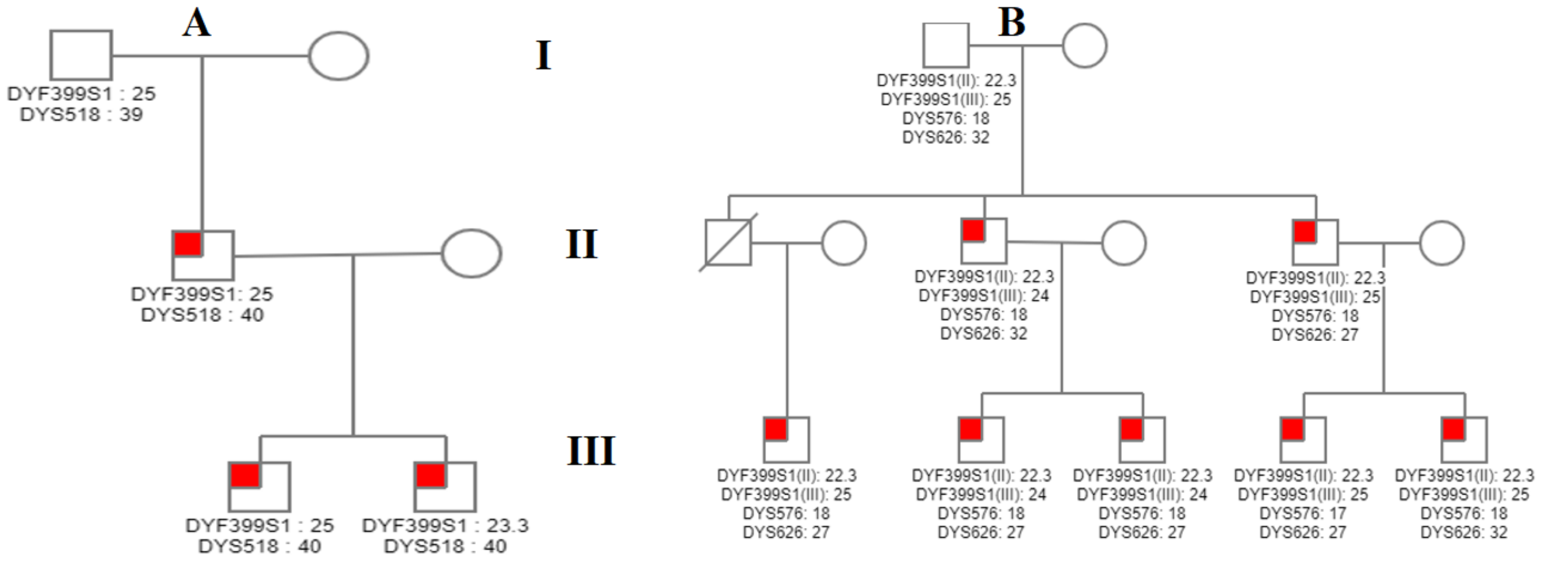

Supplementary Figure S5: (A) Paternal lineages of family ID 53 and (B) family ID 57 has been illustrated as representing mutation at one or more loci with red symbols while subtext showing the alleles.
